# Revisiting Ophidiomycosis (Snake Fungal Disease) After a Decade of Targeted Research

**DOI:** 10.3389/fvets.2021.665805

**Published:** 2021-05-31

**Authors:** Christina M. Davy, Leonard Shirose, Doug Campbell, Rachel Dillon, Christina McKenzie, Nicole Nemeth, Tony Braithwaite, Hugh Cai, Tarra Degazio, Tammy Dobbie, Sean Egan, Heather Fotherby, Jacqueline D. Litzgus, Pilar Manorome, Steve Marks, James E. Paterson, Lynne Sigler, Durda Slavic, Emily Slavik, John Urquhart, Claire Jardine

**Affiliations:** ^1^Wildlife Research and Monitoring Section, Ontario Ministry of Natural Resources, Peterborough, ON, Canada; ^2^Environmental and Life Sciences Program, Trent University, Peterborough, ON, Canada; ^3^Department of Pathobiology, University of Guelph, Guelph, ON, Canada; ^4^Canadian Wildlife Health Cooperative – Ontario/Nunavut, Guelph, ON, Canada; ^5^Southeastern Cooperative Wildlife Disease Study, University of Georgia, Athens, GA, United States; ^6^Kingsville Animal Hospital, Kingsville, ON, Canada; ^7^Animal Health Laboratory, University of Guelph, Guelph, ON, Canada; ^8^Point Pelee National Park, Leamington, ON, Canada; ^9^Egan Fife Animal Hospital, Chatham, ON, Canada; ^10^Natural Resource Solutions Inc., Waterloo, ON, Canada; ^11^Department of Biology, Laurentian University, Sudbury, ON, Canada; ^12^Ontario Parks, Ontario Ministry of Natural Resources, Peterborough, ON, Canada; ^13^Essex County Field Naturalists' Club, c/o Ojibway Nature Centre, Windsor, ON, Canada; ^14^Agriculture, Life and Environmental Sciences, University of Alberta, Edmonton, AB, Canada; ^15^Blazing Star Environmental, Oshawa, ON, Canada

**Keywords:** emerging infectious disease, fungal pathogen, ophidiomycosis, *Ophidiomyces ophidiicola*, reptile, snake, qPCR

## Abstract

Emerging infectious diseases (EIDs) are typically characterized by novelty (recent detection) and by increasing incidence, distribution, and/or pathogenicity. Ophidiomycosis, also called snake fungal disease, is caused by the fungus *Ophidiomyces ophidiicola* (formerly “*ophiodiicola”*). Ophidiomycosis has been characterized as an EID and as a potential threat to populations of Nearctic snakes, sparking over a decade of targeted research. However, the severity of this threat is unclear. We reviewed the available literature to quantify incidence and effects of ophidiomycosis in Nearctic snakes, and to evaluate whether the evidence supports the ongoing characterization of ophidiomycosis as an EID. Data from Canada remain scarce, so we supplemented the literature review with surveys for *O. ophidiicola* in the Canadian Great Lakes region. Peer-reviewed reports of clinical signs consistent with ophidiomycosis in free-ranging, Nearctic snakes date back to at least 1998, and retrospective molecular testing of samples extend the earliest confirmed record to 1986. Diagnostic criteria varied among publications (*n* = 33), confounding quantitative comparisons. Ophidiomycosis was diagnosed or suspected in 36/121 captive snakes and was fatal in over half of cases (66.7%). This result may implicate captivity-related stress as a risk factor for mortality from ophidiomycosis, but could also reflect reporting bias (i.e., infections are more likely to be detected in captive snakes, and severe cases are more likely to be reported). In contrast, ophidiomycosis was diagnosed or suspected in 441/2,384 free-ranging snakes, with mortality observed in 43 (9.8 %). Ophidiomycosis was only speculatively linked to population declines, and we found no evidence that the prevalence of the pathogen or disease increased over the past decade of targeted research. Supplemental surveys and molecular (qPCR) testing in Ontario, Canada detected *O. ophidiicola* on 76 of 657 free-ranging snakes sampled across ~136,000 km^2^. The pathogen was detected at most sites despite limited and haphazard sampling. No large-scale mortality was observed. Current evidence supports previous suggestions that the pathogen is a widespread, previously unrecognized endemic, rather than a novel pathogen. Ophidiomycosis may not pose an imminent threat to Nearctic snakes, but further research should investigate potential sublethal effects of ophidiomycosis such as altered reproductive success that could impact population growth, and explore whether shifting environmental conditions may alter host susceptibility.

## Introduction

Emerging infectious diseases are defined as diseases that have newly appeared in a population or are increasing in incidence or geographic range (1). Those affecting animals and plants (or fauna and flora) can present a major challenge to biodiversity conservation (2, 3). Fungal pathogens, in particular, are increasing in frequency (4). Impacts of fungal epidemics on biodiversity include devastating declines in American chestnut (*Castanea dentata*) populations infected with *Cryphonectria parasitica*, extinctions in some amphibian populations infected with *Batrachochytrium dendrobatidis* and *B. salamandrivorans*, and declines in some bat species infected with *Pseudogymnoascus destructans* (5). Ophidiomycosis (also called snake fungal disease) is a disease of snakes caused by *Ophidiomyces ophidiicola* [formerly “*ophiodiicola”;* Kirk (6)] (7–10). Ophidiomycosis has frequently been compared to epidemics such as bat white-nose syndrome and amphibian chytridiomycosis, and is often referred to as an emerging infectious disease that could threaten the conservation of Nearctic (North American) snakes (7, 11–16).

Ophidiomycosis occurs in wild and captive snakes in North America, Great Britain, Europe and Australia (8, 9, 17, 18) and a distinct strain of *O. ophidiicola* has been described from snakes in Europe (14). Ophidiomycosis in free-ranging snakes was first described in eastern massasauga rattlesnakes (*Sistrurus catenatus*) in North America (7), and has now been identified in snakes throughout the eastern USA, from Florida to Massachusetts to Wisconsin (8, 19–21). The fungus appears to be an opportunistic pathogen with a wide tolerance of environmental conditions (11). Experimental infection of cottonmouths (*Agkistrodon piscivorus*) and corn snakes (*Pantherophis guttatus)* with North American strains of *O. ophidiicola* caused dermal lesions consistent with those observed in wild snakes (22–24).

**Figure 1:**
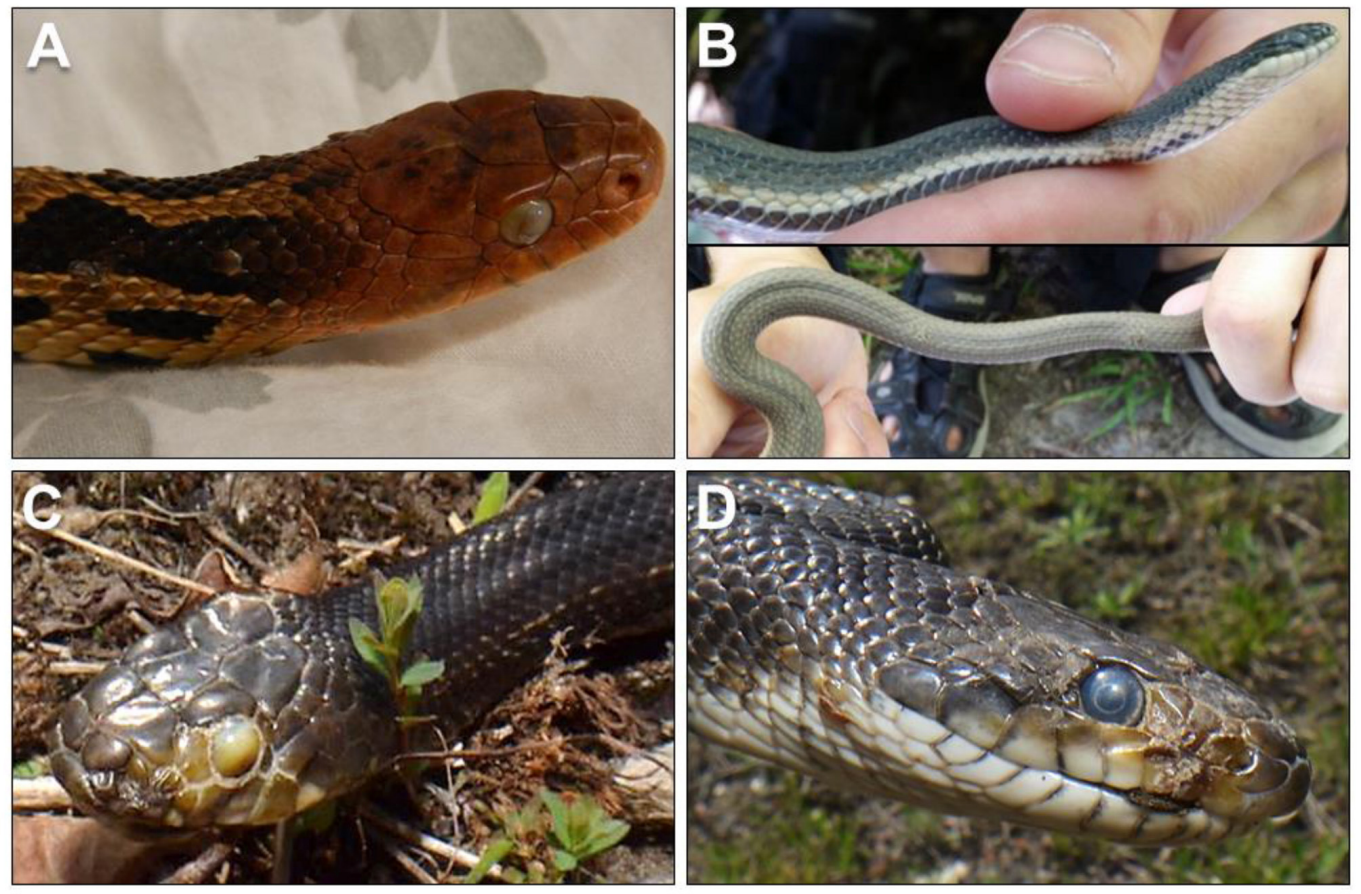
**(A)** Ophidiomycosis manifesting in gross lesions on the skin and eye of an eastern foxsnake (*Pantherophis vulpinus*) with a plaque of fungal hyphae growing over the eye (photo: Sheeva Nakhaie). **(B)** Ophidiomycosis on a queensnake (*Regina septemvittata;* photos: Heather Fotherby); **(C,D)** Gray ratsnake (*Pantherophis spiloides;* photos: Kenny Ruelland) from southeastern Ontario, Canada with suspect ophidiomycosis.

Clinical signs of ophidiomycosis range from a mild dermatitis with hyperkeratosis, scabs and crusts (Figure 1) to premature shedding of the skin, subcutaneous nodules, and corneal opacities (11). The range of clinical signs and their potential overlap with other conditions makes diagnosis difficult. Impacts to infected snakes vary with the severity of clinical signs, which can differ among individuals even in controlled, experimental infections (22, 23, 25). Facial lesions may interfere with feeding ability (22) and vision, and infection of the nasolabial pits of Viperidae could affect their heat-sensing ability, although this effect has not been directly tested. Severe cases of ophidiomycosis can end in mortality. The current standard for diagnosis of ophidiomycosis is detection of *O. ophidiicola* through real-time polymerase chain reaction [qPCR; (26)] combined with histopathological detection of fungal hyphae in lesions, particularly if diagnostic arthroconidia are present (8, 18, 27).

Research into ophidiomycosis has been mobilized by concern that *O. ophidiicola* causes or has the potential to cause widespread morbidity and mortality in free-ranging snakes (7, 11, 14–16, 23, 28). Ophidiomycosis clearly causes or contributes to mortality of snakes under some circumstances (22–25). However, it is unclear whether the available evidence indicates an impact of ophidiomycosis on the viability of snake populations (i.e., population-level effects). We reviewed the literature on ophidiomycosis in free-ranging, Nearctic snakes from a conservation ecology perspective to ask: (1) whether the available evidence supports the qualification of ophidiomycosis as an emerging infectious disease of immediate conservation concern; and (2) whether the evidence supports consideration of this disease as a current threat to populations of wild snakes. Reports of ophidiomycosis in snakes in Canada remain scarce [but see McKenzie et al. (25); Paré and Sigler (8)]. Therefore, we supplemented our literature review with a field survey of ophidiomycosis prevalence in free-ranging snakes from the Great Lakes region of Ontario, Canada, which allowed us to explore the northern distribution of the pathogen and the disease.

## Materials and Methods

The first confirmed case of ophidiomycosis from Canada was an adult, female eastern foxsnake (*Pantherophis vulpinus*) found in Point Pelee National Park, Essex County, Ontario, in late March 2015 (8). Physical examination of the snake revealed a large number of dry, crusted scabs along the dorsal midline and ventral surface of the head. The cloaca was nearly occluded by dried scabs. Skin samples and swabs were collected when the snake was taken into veterinary care. The snake underwent ecdysis while in care and the shed skin provided a further diagnostic sample. All samples were cultured for fungus at the diagnostic bacteriology laboratory of the Animal Health Laboratory (AHL) of the University of Guelph. A fungus identified as a *Chrysosporium* sp. was cultured from the skin. The isolate was confirmed to be *O. ophidiicola* by morphology and sequencing at the University of Alberta (L. Sigler) and is stored as UAMH 11863 in the UAMH Center for Global Microfungal Biodiversity (https://www.uamh.ca/index.html). Samples from this isolate were used to validate the qPCR assay conducted at the AHL.

Researchers in Canada also began to examine individuals in their study populations for signs of *O. ophidiicola* infection following the description of ophidiomycosis in free-ranging snakes in the United States (7) but different approaches were taken. Some projects in southern Ontario collected swab samples from the body surfaces of all captured snakes, regardless of whether the individuals showed signs of disease. Other projects directly swabbed only those individuals that exhibited gross lesions consistent with ophidiomycosis. Swabs were stored in lysis buffer (typically at room temperature) prior to analysis. Some projects were also able to access veterinary facilities where biopsies of gross lesions could be taken under sterile conditions, with subsequent release of the potentially affected snakes. Swabs and frozen biopsies were submitted to the Canadian Wildlife Health Cooperative (CWHC) in Guelph, Ontario for testing. From 2012 to 2016 the CWHC also received swab and tissue samples from snake carcasses submitted for diagnostic evaluation, following incidental mortalities observed in the field (predation, road mortality, or mortality with no obvious cause of death). The presence of *O. ophidiicola* on swabs or tissue samples was tested using the qPCR assay described by Allender et al. (26). Histopathology was used to investigate the presence of fungal hyphae and associated microscopic lesions in tissue samples, allowing diagnosis of ophidiomycosis following the criteria currently applied by the CWHC (Table 1). The CWHC criteria are similar to the diagnostic criteria proposed by Baker et al. (27), but do not require confirmation of arthroconidia to meet the diagnostic threshold.

**Table 1:** Criteria applied to diagnose ophidiomycosis in snakes (*n* = 657) from the Great Lakes region of Ontario, Canada.

**Presence of gross lesions**	**Histological examination of lesions detects fungal hyphae consistent with *O. ophidiicola***	***O. ophidiicola* detected through qPCR/culture**	**Diagnosis**
Yes or No	No	No	Negative
Yes or No	–	No	Not detected
–	–	Yes	Detected
Yes	–	Yes	Suspect Ophidiomycosis
Yes	Yes	–	Suspect Ophidiomycosis
–	Yes	Yes	Ophidiomycosis
Yes	Yes	Yes	Ophidiomycosis

To assess the incidence of ophidiomycosis in free-ranging, Nearctic snakes and explore whether occurrence increased over time, we searched for peer-reviewed scientific literature in Google Scholar. We used the search string [“ophidiomycosis” OR “snake fungal disease” OR “*Ophidiomyces ophiodiicola”*], and included all papers identified by this search as of 10 July 2020. We used the original spelling of the species epithet (i.e., *O. ophiodiicola*) in the search because the change to ‘*ophidiicola*' occurred very recently (6).

We reviewed all documents and retained those that provided records of ophidiomycosis in Nearctic snakes. We also accessed and reviewed publications that the initial set of studies mentioned as potential, previously unrecognized cases of ophidiomycosis. We summarized detections of the pathogen and the disease in the literature following the criteria in Table 1, which are currently used by the CWHC in Canadian surveillance of ophidiomycosis.

To assess the incidence of mortality of Nearctic snakes from ophidiomycosis, we also summarized records of mortality in the studies we reviewed. Several studies described situations in which snakes showing clinical signs of ophidiomycosis were taken into captivity for treatment. It was not possible to estimate what the outcome would have been if these snakes had been left in the wild. Other studies described mortality in free-ranging snakes that showed clinical signs of ophidiomycosis, but did not confirm the presence of the pathogen with molecular testing, culture, or histopathology. In these cases, it was not possible to be certain that the mortality was caused by ophidiomycosis. To enable an estimate of mortality incidence among studies, we assumed that all reported mortalities of snakes diagnosed with ophidiomycosis or suspected ophidiomycosis were caused by the disease. This assumption may result in an overestimate of the severity of ophidiomycosis. However, it allowed us to account for variation and related uncertainty in diagnostic criteria used among studies, and to ensure we did not inadvertently underestimate mortality associated with ophidiomycosis.

Finally, we tested whether the reported prevalence of pathogen detection (through PCR, qPCR, or cultures) or the reported prevalence of gross lesions had changed over the past decade of targeted research. We used generalized linear mixed effects models with binomial error structures, and with sampling year as a fixed effect, to test shifts in prevalence reported in the published literature (i.e., not including the additional testing we conducted on Canadian snakes, as described above). Some studies described samples collected in multiple years without reporting yearly prevalence, so we used the mean sampling year of the study for multi-year studies (e.g., samples from 2016 to 2017 = 2017.5). We scaled sampling year prior to model fitting (mean = 0, SD = 1). We included random intercepts and random slopes for species, to account for likely interspecific variation in host-pathogen interactions or environmental variation linked to species' habitat preferences.

## Results

### Detection of *O. ophidiicola* and Ophidiomycosis in Canada

**Figure 2:**
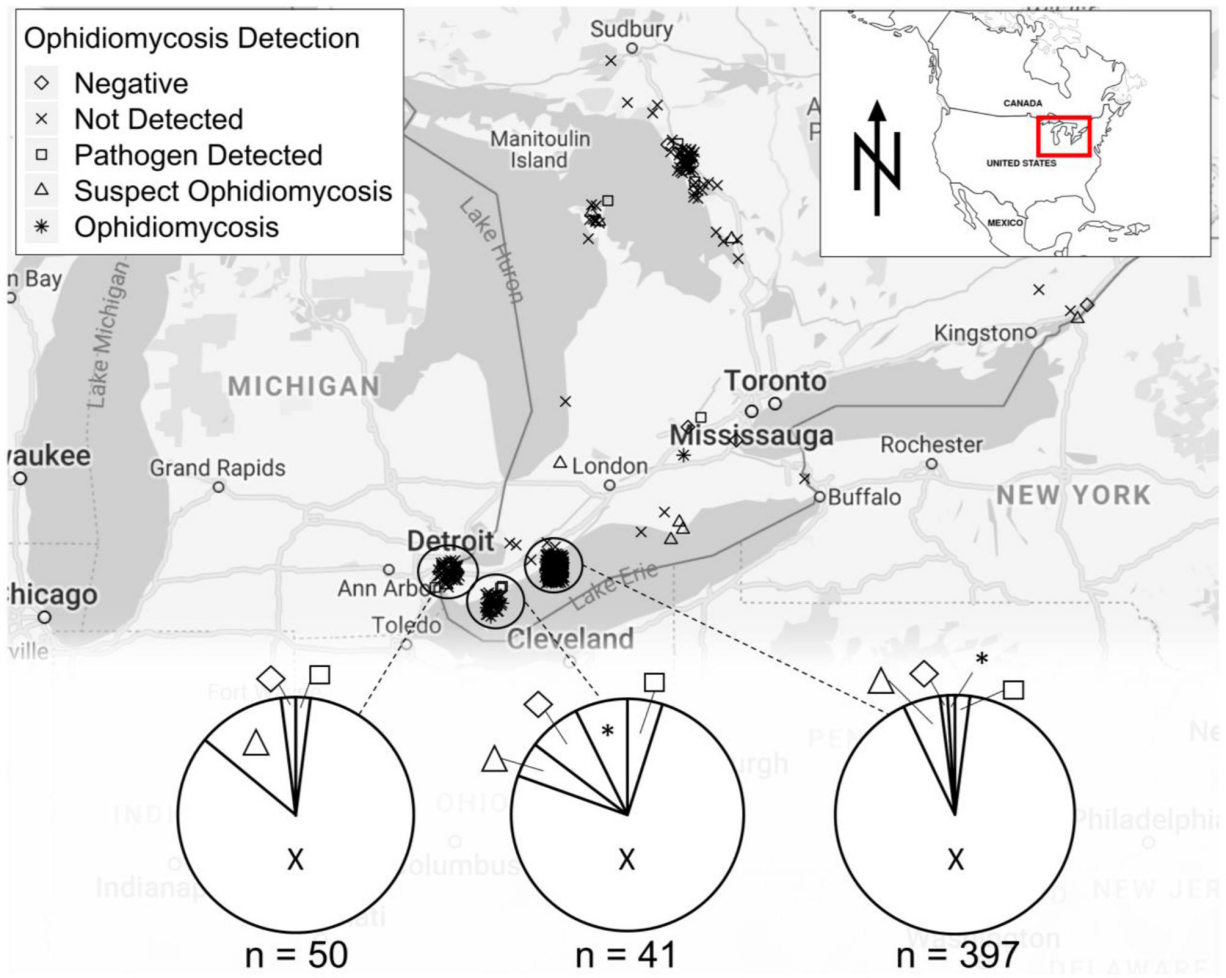
Sampling locations for snakes tested for ophidiomycosis in the Great Lakes region of Ontario, Canada, showing diagnoses based on the criteria in Table 1. Locations are shown for 542 snakes for which specific coordinates were available. A further 115 samples were tested and included in Table 2 but are not shown on the map as they were were submitted to the Canadian Wildlife Health Center without precise location data.

We collected swab or tissue samples opportunistically from 657 snakes (13 species) across southern Ontario (Figure 2). Gross lesions were observed on 116 individuals (18%, Table 2). We detected *O. ophidiicola* by qPCR on 76 snakes (11.6%), including *Nerodia sipedon, Pantherophis spiloides, P. vulpinus, Regina septemvittata, Sistrurus catenatus, Storeria dekayi*, and *Thamnophis sirtalis*. *Ophidiomyces ophidiicola* was not detected on *Heterodon platirhinos, Lampropeltis triangulum, Storeria occipitomaculata*, or *Thamnophis butleri*, but sample sizes for these species were low (Table 2). A swab from one *T. butleri* with highly suggestive lesions tested negative; a tissue sample was not available from this individual.

**Table 2:** Clinical signs of ophidiomycosis (presence of gross lesions and of hyphae in lesions by histopathology) and detection of *Ophidiomyces ophidiicola* through qPCR, from 657 snakes in southern Ontario, Canada, for which samples were submitted to the Canadian Wildlife Health Cooperative between 2012 and 2018.

		**Gross lesions present**			***O. ophidiicola*** **detected (qPCR)?**			**Presence of fungal hyphae in lesions**				**Diagnosis**			
	**Total individuals examined/sampled**	**No**	**Yes**	**Proportion of snakes with gross lesions**	**No**	**Yes**	**Proportion of swabs positive by qPCR**	**Total biopsies examined**	**No**	**Yes**	**Detected**	**Negative**	**Not detected**	**Suspect ophidiomycosis**	**Ophidiomycosis**
*Diodophis punctatus*	1	1		0	1		0						1		
*Heterodon platirhinos*	2	2		0	2		0	1	1			1	1		
*Lampropeltis triangulum*	3	3		0	3		0	2	2			2	1		
*Nerodia sipedon*	24	17	7	0.29	19	5	0.21				4		19	1	
*Pantherophis spiloides*	9	7	2	0.22	3	6	0.67	3	1	2	4	1	2		2
*Pantherophis vulpinus*	216	159	57	0.26	169	47	0.22	21	10	11	10	7	161	27	11
*Regina septemvittata*	16	1	15	0.94	7	9	0.56	1		1	1		7	7	1
*Sistrurus catenatus*	83	83		0	80	3	0.02	2	2		3	1	79		
*Storeria dekayi*	75	69	6	0.08	74	1	0.01				1		74		
*Storeria occipitomaculata*	1	1		0	1		0						1		
*Thamnophis butleri*	15	13	2	0.13	15		0	2	1	1			14	1	
*Thamnophis sauritus*	26	23	3	0.12	24	2	0.08				1		24	1	
*Thamnophis sirtalis*	186	163	23	0.12	183	3	0.02				2		183	1	
Total	657	541	116	0.18	582	76	0.12	32	16	15	26	12	567	38	14

*Some snakes were marked during mark-recapture surveys; others were sampled opportunistically and then released. It is unlikely but not impossible that some individuals were sampled more than once*.

We confirmed ophidiomycosis through a combination of histopathology and positive qPCR results in 14 snakes (11 *P. vulpinus, two P. spiloides* and one *R. septemvittata*; Table 2). Suspected ophidiomycosis was observed in 38 additional individuals. Ophidiomycosis was diagnosed as the likely cause of death in two snakes (one *P. vulpinus* and one *P. spiloides*), and two further snakes with suspected ophidiomycosis also died (one *P. vulpinus* and one *T. butleri*). A final *P. vulpinus* with ophidiomycosis died in the course of a surgery to replace an implanted radio-transmitter. Necropsy revealed that this individual also had an unusually enlarged heart and ophidiomycosis was likely not the proximate cause of death. Nevertheless, we counted this case as a mortality associated with ophidiomycosis. The other snakes with confirmed or suspected ophidiomycosis included one road-killed individual, and 44 snakes that were alive and behaving normally at the time of sampling (Table 2).

### Evaluation of Pathogen and Disease Prevalence in Wild Snakes, and Mortality in the Wild

We identified 33 peer-reviewed studies published from 2003 to July 2020 that included original observations of free-ranging, Nearctic snakes exhibiting confirmed or probable ophidiomycosis or “snake fungal disease,” or that detected *O. ophidiicola* in free-ranging, Nearctic snakes (42 species; Supplementary Table 1). The earliest observation of clinical signs consistent with ophidiomycosis that we found in the peer-reviewed literature occurred in 1997-98 (19b). However, retrospective molecular testing detected *O. ophidiicola* in a sample collected from a corn snake (*Pantherophis guttatus*) in 1986 (18).

Inferring rates of mortality associated with ophidiomycosis from the available data, we found that reported case fatality was typically high in studies that described one or several severely affected individuals. These studies also tended to describe outcomes in snakes that were taken into captivity. Summing mortalities reported in 33 peer-reviewed studies and the data from Canada reported above, ophidiomycosis was confirmed or suspected of causing the deaths of 72 individual snakes between 1999 and 2020 (Supplementary Table 1). Ophidiomycosis was diagnosed or suspected in 36/121 captive snakes and was fatal in 66.7% of cases involving snakes kept in (or brought into) captivity (Supplementary Table 1). In contrast, ophidiomycosis was diagnosed or suspected in 441/2,384 free-ranging snakes, with mortality directly observed in 43 of these individuals (9.8 %) (Supplementary Table 1).

Studies did not consistently report the same variables or apply the same diagnostic criteria (Supplementary Table 1), which limited direct comparison of prevalence, severity of clinical signs, and outcomes among populations. Nevertheless, we were able to test whether the reported prevalence of pathogen detection (through PCR, qPCR, or cultures) had changed from 2008 to 2018. Prevalence of *O. ophidiicola* reported in peer-reviewed studies declined over this time (χ^2^ = 13.86, df = 1, *P* < 0.0001). The reported prevalence of the pathogen (variance of random intercepts = 1.09) and the slope of how reported prevalence of the pathogen changed through time (variance of random slopes = 0.68) varied among the 42 species. We also tested whether the reported prevalence of gross lesions had declined from 2007 to 2018 (observations of gross lesions consistent with ophidiomycosis predated molecular detection of the pathogen). Overall, model-predicted reported prevalence of gross lesions declined between 2007 and 2018 (χ^2^ = 18.20, df = 1, *P* < 0.0001). Reported prevalence of gross lesions (variance of random intercepts = 0.54) and the slope of how reported prevalence of gross lesions changed through time (variance of random slopes = 1.69) also varied among species.

**Figure 3:**
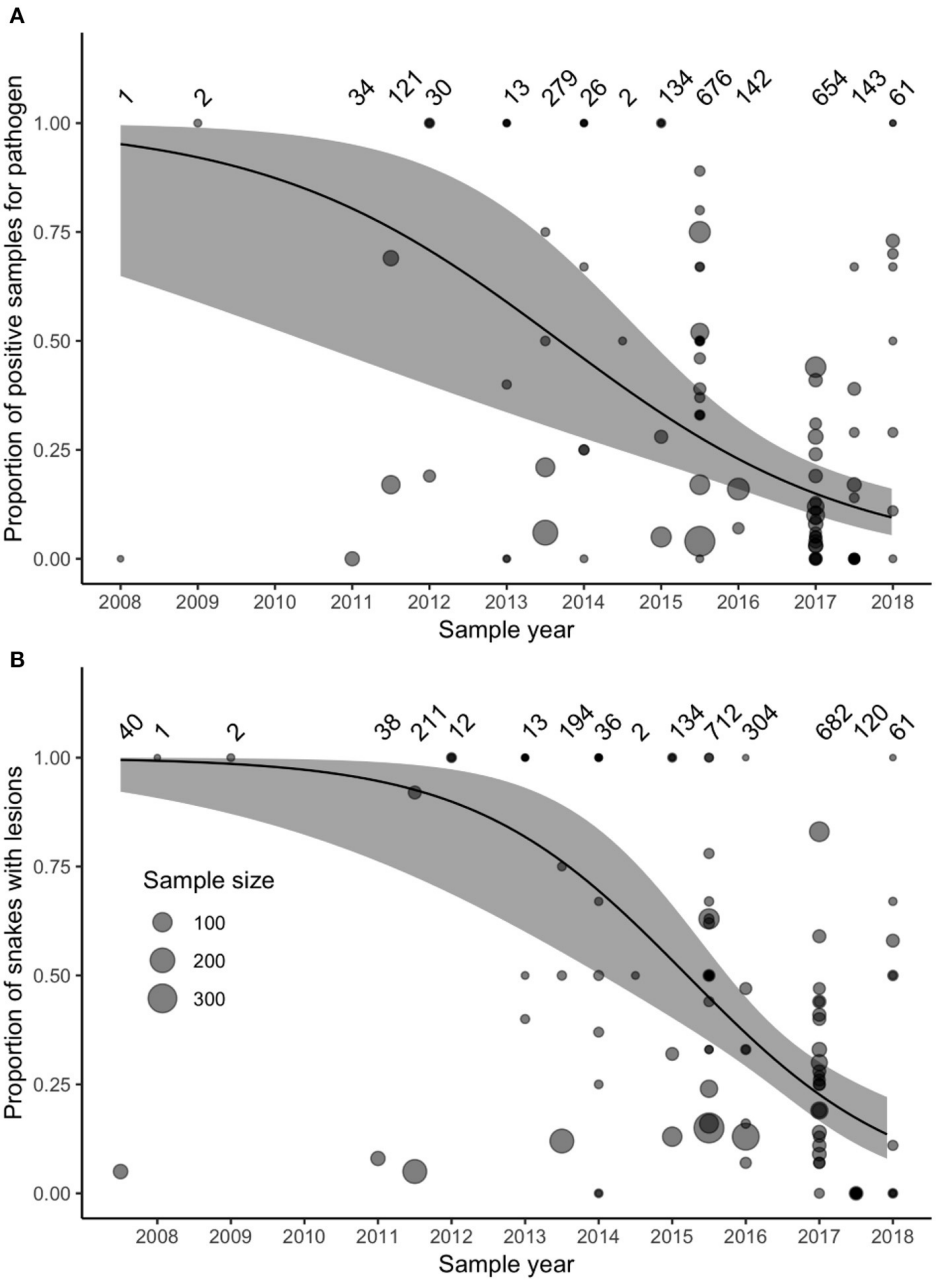
**(A)** The prevalence of *Ophidiomyces ophidiicola* (detected through PCR, qPCR, or culture) in samples collected between 2008 and 2018 and reported in the peer-reviewed literature did not increase over time. **(B)** The prevalence of reported observations of gross lesions suggestive of ophidiomycosis did not increase between 2007 and 2018. Data represent samples collected from 42 species (Supplementary Table 1). Size of points varies with sample size reported in each study, sample sizes for each time-point are noted the top of each panel, and the solid lines and gray-shaded areas indicate the fixed-effect model predictions and 95% confidence intervals.

Surveillance for *O. ophidiicola* increased substantially after 2011 (Figure 3). Our model accounted for variation in sample sizes and the number of projects collecting samples in each year, but we also reran the model including only samples collected after 2011, to ensure that our model results were not biased by increased sampling effort. This post-2011 model produced similar results to the original model that included all years.

## Discussion

The data summarized here suggest that ophidiomycosis is now most accurately understood as a relatively common but previously unrecognized disease of free-ranging Nearctic snakes, rather than as an EID of immediate conservation concern. Supporting evidence includes (1) that researchers were not looking for *O. ophidiicola* or ophidiomycosis prior to 2011; (2) that retrospective analyses confirm the fungus was present in samples collected from free-ranging snakes prior to 2011 (e.g., 9, 18); (3) that the available data do not suggest high fatality rates among free-ranging snakes with ophidiomycosis (Supplementary Table 1); (4) that the available data do not suggest an increase in the prevalence of ophidiomycosis over time, or a change in the Nearctic distribution of the fungus or disease, despite occasional observations of individual mortality potentially caused by the disease (7, 29, 30); and (5) that our field data demonstrate that low, relatively haphazard sampling effort (i.e., low number of samples collected) is sufficient to detect *O. ophidiicola* across a wide range of species and locations in southern Canada, at the northern limit of its reported Nearctic range. However, surveys with a robust, spatially explicit design would be required to truly establish the Nearctic range limits of *O. ophidiicola* and ophidiomycosis, and may still reveal an expanding distribution or shifting prevalence.

Diagnostic criteria for ophidiomycosis varied widely among the studies we reviewed (Supplementary Table 1). Some studies diagnosed “snake fungal disease” based solely on the observation of gross lesions, while others required qPCR confirmation of pathogen presence, and histopathological confirmation of fungal hyphae and/or arthroconidia in lesions. The variation in diagnostic criteria and sampling designs precludes direct comparisons of ophidiomycosis prevalence among Nearctic snakes, and highlights the importance of applying consistent criteria among studies. We also acknowledge that swabbing the skin or lesions for *O. ophidiicola* has an imperfect detection rate by qPCR (31, 32) and is generally not useful for culture. For example, swabs taken from experimentally infected cottonmouths or corn snakes did not always test positive for *O. ophidiicola* (22), and swabs of wild snakes sometimes fail to detect the fungus when it is present (32). Ideally, studies relying on swabbing to assess pathogen prevalence should estimate and report their detection accuracy. Once more standardized data are available, modeling spatio-temporal variation in disease and pathogen prevalence may reveal drivers of ophidiomycosis in free-ranging snakes, and identify vulnerable species or regions on which ophidiomycosis may have a greater impact.

When we tallied all mortalities or possible mortalities associated with ophidiomycosis reported in the literature, and the five possible mortalities reported here from our survey of snakes in southern Ontario, we found only 72 reported mortalities associated with ophidiomycosis in Nearctic snakes from 1999 to 2020 (Supplementary Table 1). This number includes snakes whose proximate causes of death were not certain. We acknowledge that most mortality in free-ranging snakes is likely to go undetected due to the cryptic behavior of many snake species, and low probability of detection for sick or dead animals in the wild. We also acknowledge that robust, longitudinal studies are required to assess potential impacts of ophidiomycosis on fecundity, and on behavior that might increase mortality risk (for example, increased basking while snakes recover could increase risk of depredation). These potential, indirect effects on survivorship deserve further research. Nevertheless, based on available reports of mortality associated with ophidiomycosis in free-ranging snakes, this disease does not appear to be devastating snake populations at this time.

Many studies of ophidiomycosis to date (including ours) focus on individuals that were selected for examination because gross lesions or other clinical signs were observed. Twenty-four of the tallied mortalities were snakes that were taken into captivity for observation, and died in captivity. Outcomes in captivity may not reflect survivorship in free-ranging, infected snakes, and the apparent association between captivity and ophidiomycosis-associated mortality suggests that stress associated with captivity may exacerbate the effects of the disease (33). Furthermore, ophidiomycosis appears to have a high case fatality rate in cases with severe clinical signs, but this conclusion relies heavily on case studies with very small sample sizes [e.g., see Table 1 in Lorch et al. (15)] that do not allow estimates of per capita mortality rates from ophidiomycosis in affected, free-ranging populations. The most robust published survey on the prevalence of potential ophidiomycosis in a wild population (19) reports fungal dermatitis and stomatitis in 9.8% of captured *Sistrurus miliarius barbouri* (> 10,000 captures of > 600 individuals). The pathogen involved was not confirmed by culture or qPCR, but a histopathology image showing arthroconidia erumpent from a skin lesion clearly identifies *O. ophidiicola* (18, 19). No mortalities were associated with the disease, but other effects (e.g., on fecundity or behavior) were not explored. More recently, declines in a population of *C. horridus* in New Hampshire were tentatively associated with observations of a fungal disease in some individuals (29). This study is frequently cited as evidence for population-level effects of ophidiomycosis, and we counted these cases as ophidiomycosis-associated mortalities in our summary, in keeping with our conservative approach. However, we note that Clark et al. (29) did not identify the pathogen, and attributed the observed mortality to a combination of inbreeding, disease, and extreme climate events, not unequivocally to disease. We found no other evidence of likely population declines associated with ophidiomycosis.

Multiple factors may determine the impacts of ophidiomycosis on free-ranging snakes (15). We acknowledge that radio-telemetry studies in ophidiomycosis-exposed populations should be carried out cautiously to ensure that attachment or implantation of transmitters does not increase susceptibility to ophidiomycosis (7, 30). Nevertheless, the impact of ophidiomycosis on individual fitness in free-ranging snakes can only be evaluated by comparing the survivorship, behavior and reproductive output of infected and uninfected individuals in their natural habitats (34, 35). Research on population-level effects of ophidiomycosis will benefit from future studies clearly reporting the following information, where possible: (1) the total number of free-ranging snakes examined, and the survey method; (2) the number of snakes observed with gross lesions; (3) the number of snakes with and without gross lesions that were swabbed and/or biopsied, (4) the number of these samples that tested positive for *O. ophidiicola* using qPCR*; (*5) the number of samples for which histopathology and qPCR confirmed a diagnosis of ophidiomycosis, and (6) the specific diagnostic criteria used.

Our results confirm the presence of *O. ophidiicola* and ophidiomycosis in several snake species across a wide geographic area in Ontario and extend the known distribution of the pathogen substantially northward (Figure 2). Some snake species in which *O. ophidiicola* was detected are listed as species at risk in Canada, including *P. spiloides, P. vulpinus*, and *S. catenatus*. If ophidiomycosis poses a current or future threat to population persistence, then the presence of the disease is a conservation concern for Canadian snakes. Fortunately, the available evidence does not suggest that it is causing substantial mortality of free-ranging snakes in Canada at this time. However, ongoing surveillance of ophidiomycosis in free-ranging snakes is advised, as rapidly shifting environmental conditions may alter host susceptibility to wildlife diseases.

Evidence from long-term studies indicates that global declines of many reptile species are severe and broad in terms of both geographic scope and range of taxa affected (15). In a study of 17 snake populations in the UK, France, Italy, Nigeria, and Australia between 1995 and 2009, two-thirds of the monitored populations collapsed and have shown no signs of recovery over the nearly a decade following the populations' declines (36). In Canada, COSEWIC lists 18 of the 33 distinct subspecies of snake as being at risk (http://www.cosewic.gc.ca/). The cause of any specific decline can be a complex interaction of cumulative and sometimes synergistic factors. Globally, reptile populations declines are driven by a combination of threats, including habitat loss and degradation, introduced invasive species, environmental pollution, global climate change, unsustainable use and persecution, and disease and parasitism (37–39). In this framework, the emergence of a widespread and virulent infectious disease could exacerbate existing population declines. However, the data summarized here do not support the prioritization of ophidiomycosis as a conservation crisis. We did not find evidence for population-level impacts of ophidiomycosis on Nearctic snakes. Indeed, individuals in some populations of *S. catenatus* and eastern indigo snakes (*Drymarchon couperi*) appear to tolerate ophidiomycosis (32, 40), and free-ranging *P. vulpinus* with relatively severe clinical signs of ophidiomycosis that are left in the wild can often resolve their clinical signs with no direct effect on fitness (34). Nevertheless, the interactions between ophidiomycosis and other pressures (i.e., syndemics) merit further research, as diseases can interact with other threats such as climate change and landscape modification to exacerbate population declines (41, 42).

## Data Availability Statement

The raw data supporting the conclusions of this article will be made available by the authors, without undue reservation.

## Ethics Statement

The animal study was reviewed and approved by Ontario Ministry of Natural Resources and Forestry, Wildlife Animal Care Committee.

## Author Contributions

CD and LSh conceived the study. CD, RD, TB, TDe, TDo, SE, HF, JL, PM, SM, JP, ES, and JU collected samples from wild snakes for diagnostic testing. LSi identified the pathogen on its first documented appearance in Canada, and provided mycology expertise to the study. LSh, DC, CM, NN, and CJ assessed and diagnosed specimens. HC and DS conducted qPCR assays to detect the pathogen. RD, JP, CM, and CD conducted the literature review. RD and JP conducted statistical analyses and generated maps of pathogen distribution. CD, LSh, and LSi led the writing of the manuscript. All authors edited and approved the manuscript prior to submission.

## Conflict of Interest

HF was employed by the company Natural Resource Solutions Incorporated. The remaining authors declare that the research was conducted in the absence of any commercial or financial relationships that could be construed as a potential conflict of interest.
