# Supplementary material for: Revisiting Ophidiomycosis (Snake Fungal Disease) After a Decade of Targeted Research
Source: Front Vet Sci. 2021 May 31;8:665805. doi: 10.3389/fvets.2021.665805 (PMC8200636; doi:10.3389/fvets.2021.665805)
Supplement: Supplementary file 1 [file Table_1.docx]

**Supplementary Table 1.** Free-ranging, Nearctic snakes reported in peer-reviewed literature with suspected or confirmed presence of *Ophidiomyces ophidiicola* and/or cases of ophidiomycosis, and outcomes or suspected outcomes of these cases. In the “species” column, species in **bold** are free-ranging, and un-bolded species indicate the snakes were originally free-ranging or captive, but were kept in captivity for the purposes of the described observations of ophidiomycosis. “Year” indicates the year(s) the samples were collected (not the publication year).

| **Species** | **Citation** | **Year** | **# snakes examined/ sampled** | **Presence of gross lesions?*** | | | **qPCR/PCR/culture detection of *Ophidiomyces ophidiicola?*** | | | | **Presence of fungal hyphae in lesions?** | | **Diagnosis** | | | | |  |
| --- | --- | --- | --- | --- | --- | --- | --- | --- | --- | --- | --- | --- | --- | --- | --- | --- | --- | --- |
|  |  |  |  | **No** | **Yes** | **Proportion with gross lesions** | **No** | **Yes** | **Proportion of samples positive with qPCR** | **Total samples examined** | **No** | **Yes** | **Negative** | **Not Detected** | **Detected** | **Suspected Ophidiomycosis** | **Ophidiomycosis** | **Mortalities associated with ophidiomycosis** |
| ***Agkistrodon contortrix*** | (McKenzie et al., 2019) | 2015–2016 | 18 | 9 | 9 | 0.5 | 11 | 7 | 0.39 | NA | NA | NA | 0 | 9 | 0 | 7 | 0 | 0 |
| ***Agkistrodon contortrix*** | (Lorch et al., 2016) | 2013 | 1 | 0 | 1 | 1 | 0 | 1 | 1 | 1 | 0 | 1 | 0 | 0 | 0 | 0 | 1 | 1 |
| ***Agkistrodon contortrix*** | (Snyder and Sutton, 2020) | 2017–2018 | 32 | 32 | 0 | 0 | 26 | 6 | 0.17 | NA | NA | NA | 0 | 26 | 6 | 0 | 0 | 0 |
| ***Agkistrodon contortrix*** | (Haynes et al. 2020) | 2016–2018 | 13 | 11 | 2 | 0.15 | 12 | 1 | 0.08 | NA | NA | NA | 0 | 12 | 0 | 1 | 0 | 0 |
| ***Agkistrodon piscivorus*** | (Snyder and Sutton, 2020) | 2017–2018 | 3 | 3 | 0 | 0 | 3 | 0 | 0 | NA | NA | NA | 0 | 3 | 0 | 0 | 0 | 0 |
| ***Agkistrodon piscivorus*** | (Haynes et al. 2020) | 2016–2018 | 39 | 26 | 13 | 0.33 | 28 | 11 | 0.28 | NA | NA | NA | 0 | 28 | 0 | 11 | 0 | 0 |
| ***Carphophis amoenus*** | (Fuchs et al., 2020) | 2018 | 7 | 7 | 0 | 0 | 5 | 2 | 0.29 | NA | NA | NA | 0 | 5 | 2 | 0 | 0 | 0 |
| ***﻿Carphophis amoenus*** | (McKenzie et al., 2019) | 2015–2016 | 6 | 2 | 4 | 0.67 | 2 | 4 | 0.67 | NA | NA | NA | 0 | 2 | 0 | 4 | 0 | 0 |
| ***Carphophis amoenus*** | (Snyder and Sutton, 2020) | 2017–2018 | 2 | 2 | 0 | 0 | 2 | 0 | 0 | NA | NA | NA | 0 | 2 | 0 | 0 | 0 | 0 |
| ***Cemophora coccinea*** | (Snyder and Sutton, 2020) | 2017–2018 | 1 | 1 | 0 | 0 | 1 | 0 | 0 | NA | NA | NA | 0 | 1 | 0 | 0 | 0 | 0 |
| ***Cemophora coccinea*** | (Haynes et al. 2020) | 2016–2018 | 28 | 25 | 3 | 0.11 | 27 | 1 | 0.04 | NA | NA | NA | 0 | 27 | 0 | 1 | 0 | 0 |
| ***Coluber constrictor*** | (Lorch et al., 2016) | 2012­–2015 | 6 | 4 | 2 | 0.5 | 4 | 2 | 0.33 | 6 | 2 | 4 | 2 | 0 | 0 | 2 | 2 | 2 |
| ***Coluber constrictor*** | (Guthrie et al., 2016) | 2014 | 3 | 1 | 2 | 0.67 | 1 | 2 | 0.67 | 2 | 0 | 2 | 0 | 0 | 0 | 2 | 0 | 0 |
| ***Coluber constrictor*** | (Fuchs et al., 2020) | 2018 | 3 | 1 | 2 | 0.67 | 1 | 2 | 0.67 | NA | NA | NA | 0 | 1 | 0 | 2 | 0 | 0 |
| *Coluber constrictor* | (Hill et al., 2018) | 2015–2016 | 4 | 2 | 2 | 0.5 | 2 | 2 | 0.5 | 1 | 0 | 1 | 2 | 1 | 0 | 0 | 1 | 1 (Euthanized) |
| ***﻿Coluber constrictor*** | (McKenzie et al., 2019) | 2015–2016 | 13 | 5 | 8 | 0.62 | 7 | 6 | 0.46 | NA | NA | NA | 0 | 4 | 1 | 5 | 0 | 0 |
| ***Coluber constrictor*** | (Snyder and Sutton, 2020) | 2017–2018 | 1 | 1 | 0 | 0 | 1 | 0 | 0 | NA | NA | NA | 0 | 1 | 0 | 0 | 0 | 0 |
| ***Coluber constrictor*** | (Haynes et al. 2020) | 2016–2018 | 57 | 40 | 17 | 0.30 | 50 | 7 | 0.12 | NA | NA | NA | 0 | 50 | 0 | 7 | 0 | 0 |
| ***Coluber constrictor constrictor*** | (Licitra et al., 2019)**††††** | 2015, 2017 | 9 | 6 | 3 | 0.33 | NR | NR | NR | NR | NR | NR | 0 | 0 | 0 | 3 | 0 | 0 |
| ***Coluber flagellum*** | (Haynes et al. 2020) | 2016–2018 | 25 | 15 | 10 | 0.40 | 25 | 0 | 0 | NA | NA | NA | 0 | 25 | 0 | 0 | 0 | 0 |
| ***Crotalus adamanteus*** | (Haynes et al. 2020) | 2016–2018 | 25 | 18 | 7 | 0.28 | 19 | 6 | 0.24 | NA | NA | NA | 0 | 19 | 0 | 6 | 0 | 0 |
| ***Crotalus horridus*** | (Clark et al., 2011)† | 2006–2009 | 40 | 38 | 2 | 0.05 | NA | NA | NA | NA | NA | NA | NA | NA | NA | NA | NA | 20 |
| *Crotalus horridus* | (McBride et al., 2015) | 2011–2013 | 8 | 0 | 8 | 1 | 0 | 8 | 1 | NA | NA | NA | 0 | 0 | 0 | 8 | 0 | 1 |
| ***Crotalus horridus*** | (Lorch et al., 2016) | 2008–2015 | 26 | 2 | 24 | 0.92 | 26 | 18 | 0.69 | 26 | 2 | 24 | 2 | 0 | 0 | 6 | 18 | 2 |
| ***Crotalus horridus*** | (Stengle, 2018) | 2009–2014 | 185 | 176 | 9 | 0.05 | NA | NA | NA | NA | NA | NA | NA | 0 | 0 | 0 | 0 | 0 |
| *Crotalus horridus* | (Britton et al., 2019) | 2016 | 14 (13 born in captivity) | 13 | 1 | 0.07 | 13 | 1 | 0.07 | 2 | 2 | 0 | 2 | 11 | 0 | 1 | 0 | 0 |
| *Crotalus horridus* | (Hill et al., 2018) | 2015–2016 | 8 | 4 | 4 | 0.5 | 5 | 3 | 0.37 | 1 | 1 | 0 | 5 | 2 | 0 | 1 | 0 | 0 |
| ***﻿Crotalus horridus*** | (McKenzie et al., 2019) | 2015–2016 | 3 | 0 | 3 | 1 | 1 | 2 | 0.67 | NA | NA | NA | 0 | 0 | 0 | 2 | 0 | 0 |
| ***Crotalus horridus*** | (Snyder and Sutton, 2020) | 2017–2018 | 3 | 3 | 0 | 0 | 3 | 0 | 0 | NA | NA | NA | 0 | 3 | 0 | 0 | 0 | 0 |
| ***Crotalus horridus*** | (Haynes et al. 2020) | 2016–2018 | 15 | 14 | 1 | 0.07 | 13 | 2 | 0.13 | NA | NA | NA | 0 | 13 | 0 | 2 | 0 | 0 |
| ***Diadophis punctatus*** | (Fuchs et al., 2020) | 2018 | 1 | 1 | 0 | 0 | 0 | 1 | 1 | NA | NA | NA | 0 | 0 | 1 | 0 | 0 | 0 |
| ***﻿Diadophis punctatus*** | (McKenzie et al., 2019) | 2015–2016 | 9 | 5 | 4 | 0.44 | 6 | 3 | 0.33 | NA | NA | NA | 0 | 5 | 0 | 3 | 0 | 0 |
| ***Diadophis punctatus*** | (Snyder and Sutton, 2020) | 2017–2018 | 5 | 5 | 0 | 0 | 5 | 0 | 0 | NA | NA | NA | 0 | 5 | 0 | 0 | 0 | 0 |
| ***Diadophis punctatus*** | (Haynes et al. 2020) | 2016–2018 | 11 | 8 | 3 | 0.27 | 10 | 1 | 0.09 | NA | NA | NA | 0 | 10 | 0 | 1 | 0 | 0 |
| ***Drymarchon couperi*** | (Chandler et al., 2019) | 2016–2018 | 107 | 18 | 89 | 0.83 | 59 | 47 | 0.44 | NA | NA | NA | 0 | 59 | 0 | 46 | 0 | 0 |
| *Elaphe obsoleta obsoleta* | (Rajeev et al., 2009) | UNK | 1 | 0 | 1 | 1 | 0 | 1 | 1 | 1 | 0 | 1 | 0 | 0 | 0 | 0 | 1 | 1 (Died in captivity) |
| *Farancia abacura* | (Last et al., 2016) | 2014 | 1 | 0 | 1 | 1 | 0 | 1 | 1 | 1 | 0 | 1 | 0 | 0 | 0 | 0 | 1 | 1 (Euthanized) |
| ***Farancia abacura*** | (Haynes et al. 2020) | 2016–2018 | 27 | 15 | 12 | 0.44 | 22 | 5 | 0.19 | NA | NA | NA | 0 | 22 | 0 | 5 | 0 | 0 |
| ***Farancia erytrogramma*** | (Guthrie et al., 2016) | 2014 | 1 | 0 | 1 | 1 | 0 | 1 | 1 | 1 | 0 | 1 | 0 | 0 | 0 | 1 | 0 | 0 |
| ***Heterodon nasicus*** | (Lorch et al., 2016) | 2014 | 2 | 0 | 2 | 1 | 2 | 0 | 0 | 2 | 0 | 2 | 0 | 0 | 0 | 2 | 0 | 0 |
| ***Heterodon platirhinos*** | (Haynes et al. 2020) | 2016–2018 | 35 | 30 | 5 | 0.14 | 34 | 1 | 0.03 | NA | NA | NA | 0 | 34 | 0 | 1 | 0 | 0 |
| ***Lampropeltis calligaster*** | (Snyder and Sutton, 2020) | 2017–2018 | 1 | 1 | 0 | 0 | 1 | 0 | 0 | NA | NA | NA | 0 | 1 | 0 | 0 | 0 | 0 |
| ***Lampropeltis elapsoides*** | (Haynes et al. 2020) | 2016–2018 | 8 | 8 | 0 | 0 | 8 | 0 | 0 | NA | NA | NA | 0 | 8 | 0 | 0 | 0 | 0 |
| ***Lampropeltis getula*** | (Fuchs et al., 2020) | 2018 | 1 | 0 | 1 | 1 | 0 | 1 | 1 | NA | NA | NA | 0 | 0 | 0 | 1 | 0 | 0 |
| ***﻿Lampropeltis getula*** | (McKenzie et al., 2019) | 2015–2016 | 9 | 2 | 7 | 0.78 | 1 | 8 | 0.89 | NA | NA | NA | 0 | 1 | 1 | 7 | 0 | 0 |
| ***Lampropeltis getula*** | (Haynes et al. 2020) | 2016–2018 | 16 | 12 | 4 | 0.25 | 11 | 5 | 0.31 | NA | NA | NA | 0 | 11 | 0 | 5 | 0 | 0 |
| ***Lampropeltis getula getula*** | (Guthrie et al., 2016) | 2014 | 1 | 1 | 0 | 0 | NA | NA | NA | NA | NA | NA | NA | 0 | 0 | 0 | 0 | 0 |
| ***Lampropeltis nigra*** | (Lorch et al., 2016) | 2013 | 1 | 0 | 1 | 1 | 0 | 1 | 1 | 1 | 0 | 1 | 0 | 0 | 0 | 0 | 1 | 1 |
| ***Lampropeltis nigra*** | (Snyder and Sutton, 2020) | 2017–2018 | 7 | 7 | 0 | 0 | 5 | 2 | 0.29 | NA | NA | NA | 0 | 5 | 2 | 0 | 0 | 0 |
| ***Lampropeltis triangulum*** | (Lorch et al., 2016) | 2013–2014 | 4 | 1 | 3 | 0.75 | 1 | 3 | 0.75 | 4 | 1 | 3 | 1 | 0 | 0 | 0 | 3 | 2 |
| ***Lampropeltis triangulum*** | (Ravesi et al., 2016) | 2015 | 1 | 0 | 1 | 1 | 0 | 1 | 1 | NA | NA | NA | 0 | 0 | 0 | 1 | 0 | 0 |
| ***Lampropeltis Triangulum*** | (McKenzie et al., 2019) | 2015–2016 | 5 | 0 | 5 | 1 | 1 | 4 | 0.8 | NA | NA | NA | 0 | 0 | 0 | 4 | 0 | 0 |
| ***Lampropeltis triangulum*** | (Snyder and Sutton, 2020) | 2017–2018 | 2 | 2 | 0 | 0 | 2 | 0 | 0 | NA | NA | NA | 0 | 2 | 0 | 0 | 0 | 0 |
| ***Liodytes pygaea*** | (Haynes et al. 2020) | 2016–2018 | 15 | 14 | 1 | 0.07 | 15 | 0 | 0 | NA | NA | NA | 0 | 15 | 0 | 0 | 0 | 0 |
| ***Liodytes rigida*** | (Haynes et al. 2020) | 2016–2018 | 20 | 15 | 5 | 0.25 | 20 | 0 | 0 | NA | NA | NA | 0 | 20 | 0 | 0 | 0 | 0 |
| ***﻿Nerodia erythrogaster*** | (McKenzie et al., 2019) | 2015–2016 | 2 | 1 | 1 | 0.5 | 1 | 1 | 0.5 | NA | NA | NA | 0 | 1 | 0 | 1 | 0 | 0 |
| ***Nerodia erythrogaster*** | (Snyder and Sutton, 2020) | 2017–2018 | 2 | 2 | 0 | 0 | 2 | 0 | 0 | NA | NA | NA | 0 | 2 | 0 | 0 | 0 | 0 |
| ***Nerodia erythrogaster*** | (Haynes et al. 2020) | 2016–2018 | 27 | 11 | 16 | 0.59 | 16 | 11 | 0.41 | NA | NA | NA | 0 | 16 | 0 | 11 | 0 | 0 |
| *Nerodia erythrogaster transversa* | (Barber et al., 2016) | 2013 | 1 | 0 | 1 | 1 | 1 | 0 | 0 | 1 | 0 | 1 | 0 | 0 | 0 | 1 | 0 | 1 (Euthanized) |
| *Nerodia fasciata* | (Haynes et al. 2020) | 2016–2018 | 69 | 56 | 13 | 0.19 | 62 | 7 | 0.10 | NA | NA | NA | 0 | 62 | 0 | 7 | 0 | 0 |
| *Nerodia sipedon* | (Glorioso et al., 2016) | 2015 | 1 | 0 | 1 | 1 | 0 | 1 | 1 | 1 | 0 | 1 | 0 | 0 | 0 | 0 | 1 | 1 (Euthanized) |
| ***Nerodia sipedon*** | (Lorch et al., 2016) | 2012–2014 | 1 | 0 | 1 | 1 | 0 | 1 | 1 | 1 | 0 | 1 | 0 | 0 | 0 | 0 | 1 | 1 |
| ***Nerodia sipedon*** | (Guthrie et al., 2016) | 2014 | 8 | 5 | 3 | 0.37 | 6 | 2 | 0.25 | 3 | 0 | 2 | NA | 0 | 0 | 2 | 0 | 0 |
| ***Nerodia sipedon*** | (Fuchs et al., 2020) | 2018 | 26 | 11 | 15 | 0.58 | 7 | 19 | 0.73 | NA | NA | NA | 0 | 7 | 0 | 19 | 0 | 0 |
| ***Nerodia sipedon*** | (McKenzie et al., 2019) | 2015–2016 | 72 | 55 | 17 | 0.24 | 34 | 38 | 0.52 | NA | NA | NA | 0 | 34 | 21 | 17 | 0 | 0 |
| ***Nerodia sipedon*** | (Snyder and Sutton, 2020) | 2017–2018 | 3 | 3 | 0 | 0 | 1 | 2 | 0.67 | NA | NA | NA | 0 | 1 | 2 | 0 | 0 | 0 |
| ***Nerodia sipedon insularis*** | (Lorch et al., 2016) | 2009 | 2 | 0 | 2 | 1 | 0 | 2 | 1 | 2 | 0 | 2 | 0 | 0 | 0 | 0 | 2 | 2 |
| ***Nerodia sipedon sipedon*** | (Licitra et al., 2019)**††††** | 2015, 2017 | 19 | 10 | 9 | 0.47 | NR | NR | NR | NR | NR | NR | 0 | 0 | 0 | 9 | 0 | 0 |
| ***Nerodia taxispilota*** | (Guthrie et al., 2016) | 2014 | 8 | 4 | 4 | 0.5 | 6 | 2 | 0.25 | 2 | 1 | 1 | 1 | 0 | 0 | 0 | 1 | 0 |
| ***Nerodia taxispilota*** | (Haynes et al. 2020) | 2016–2018 | 33 | 18 | 15 | 0.45 | 18 | 15 | 0.45 | NA | NA | NA | 0 | 18 | 0 | 15 | 0 | 0 |
| ***﻿Opheodrys aestivus*** | (McKenzie et al., 2019) | 2015–2016 | 2 | 1 | 1 | 0.5 | 2 | 0 | 0 | NA | NA | NA | 0 | 1 | 0 | 0 | 0 | 0 |
| ***Opheodrys aestivus*** | (Snyder and Sutton, 2020) | 2017–2018 | 2 | 2 | 0 | 0 | 2 | 0 | 0 | NA | NA | NA | 0 | 2 | 0 | 0 | 0 | 0 |
| ***Opheodrys aestivus*** | (Haynes et al. 2020) | 2016–2018 | 19 | 10 | 9 | 0.47 | 18 | 1 | 0.05 | NA | NA | NA | 0 | 18 | 0 | 1 | 0 | 0 |
| ***Pantherophis alleghaniensis*** | (Licitra et al., 2019)**††††** | 2015, 2017 | 6 | 5 | 1 | 0.16 | NR | NR | NR | NR | NR | NR | 0 | 0 | 0 | 1 | 0 | 0 |
| ***Pantherophis alleghaniensis*** | (Lorch et al., 2016) | 2011–2015 | 5 | 3 | 2 | 0.4 | 3 | 2 | 0.4 | 5 | 3 | 2 | 3 | 0 | 0 | 0 | 2 | 1 |
| ***Pantherophis alleghaniensis*** | (Guthrie et al., 2016) | 2014 | 4 | 3 | 1 | 0.25 | NA | NA | NA | 1 | 0 | 1 | NA | 0 | 0 | 1 | 0 | 0 |
| ***Pantherophis alleghaniensis*** | (Fuchs et al., 2020) | 2018 | 10 | 5 | 5 | 0.5 | 3 | 7 | 0.7 | NA | NA | NA | 0 | 3 | 0 | 7 | 0 | 0 |
| ***Pantherophis alleghaniensis*** | (Haynes et al. 2020) | 2016–2018 | 61 | 37 | 24 | 0.38 | 53 | 8 | 0.13 | NA | NA | NA | 0 | 53 | 0 | 8 | 0 | 0 |
| ***Pantherophis guttatus*** | (Snyder and Sutton, 2020) | 2017–2018 | 1 | 1 | 0 | 0 | 1 | 0 | 0 | NA | NA | NA | 0 | 1 | 0 | 0 | 0 | 0 |
| ***Pantherophis guttatus*** | (Haynes et al. 2020) | 2016–2018 | 32 | 29 | 3 | 0.09 | 31 | 1 | 0.03 | NA | NA | NA | 0 | 31 | 0 | 1 | 0 | 0 |
| ***﻿Pantherophis spiloides*** | (McKenzie et al., 2019) | 2015–2016 | 2 | 1 | 1 | 0.5 | 1 | 1 | 0.5 | NA | NA | NA | 0 | 1 | 0 | 1 | 0 | 0 |
| ***Pantherophis spiloides*** | (Snyder and Sutton, 2020) | 2017–2018 | 10 | 10 | 0 | 0 | 10 | 0 | 0 | NA | NA | NA | 0 | 10 | 0 | 0 | 0 | 0 |
| ***Pantherophis vulpinus*** | (Lorch et al., 2016) | 2013–2015 | 2 | 0 | 2 | 1 | 0 | 2 | 1 | 2 | 0 | 2 | 0 | 0 | 0 | 0 | 2 | 2 |
| ***Pituophis catenifer sayi*** | (Lorch et al., 2016) | 2012–2014 | 2 | 1 | 1 | 0.5 | 2 | 0 | 0 | 2 | 1 | 1 | 1 | 0 | 0 | 1 | 0 | 0 |
| ***Pituophis melanoleucus*** | (Haynes et al. 2020) | 2016–2018 | 9 | 5 | 4 | 0.44 | 8 | 1 | 0.11 | NA | NA | NA | 0 | 8 | 0 | 1 | 0 | 0 |
| ***Python bivittatus*** | (Lorch et al., 2016) | 2008 | 1 | 0 | 1 | 1 | 1 | 0 | 0 | 1 | 0 | 1 | 0 | 0 | 0 | 0 | 1 | 1 |
| ***Regina septemvittata*** | (Lorch et al., 2016) | 2014–2015 | 2 | 1 | 1 | 0.50 | 1 | 1 | 0.50 | 2 | 1 | 1 | 1 | 0 | 0 | 0 | 1 | 1 |
| ***Regina septemvittata*** | (Price et al., 2016) | 2014 | 1 | 0 | 1 | 1 | 0 | 1 | 1 | 1 | 0 | 1 | 0 | 0 | 0 | 0 | 1 | 1 |
| ***Regina septemvittata*** | (McKenzie et al., 2019) | 2015–2016 | 116 | 43 | 73 | 0.63 | 29 | 87 | 0.75 | NA | NA | NA | 0 | 17 | 26 | 61 | 0 | 0 |
| *Sistrurus catenatus* | (Allender et al., 2011) | 2008 | NR | NR | 4 | NA | NR | 4 | NA | NR | NR | 4 | 0 | 0 | 0 | 0 | 4 | 3 |
| ***Sistrurus catenatus*** | (Allender et al., 2013) | 2011 | 38 | 35 | 3 | 0.08 | 34 | 0 | 0 | NA | NA | NA | 0 | 34 | 0 | 0 | 0 | 0 |
| ***Sistrurus catenatus*** | (Allender et al., 2016a) | 2015 | 102 | 89 | 13 | 0.13 | 97 | 5 | 0.05 | NA | NA | NA | 0 | 97 | 0 | 5 | 0 | 0 |
| ***Sistrurus catenatus*** | (Lorch et al., 2016) | 2015 | 5 | 0 | 5 | 1 | 0 | 5 | 1 | 5 | 0 | 5 | 0 | 0 | 0 | 0 | 5 | 0 |
| *Sistrurus catenatus* | (Tetzlaff et al., 2015) | 2013 | 2 | 0 | 2 | 1 | 0 | 2 | 1 | 2 | 0 | 2 | 0 | 0 | 0 | 0 | 2 | 2 (Died in captivity) |
| ***Sistrurus catenatus*** | (Allender et al., 2018) | 2015–2016 | 96 (6 snakes retested) | 81 | 15 | 0.16 | 80 | 16 | 0.17 | NA | NA | NA | 0 | 80 | 0 | 16 | 0 | 0 |
| ***Sistrurus catenatus*** | (Hileman et al., 2018) | 2014–2016 | 297 | 289 | 44 | 0.15 | 284 | 13 | 0.04 | NA | NA | NA | 0 | 284 | 0 | 13 | 0 | 0 |
| *Sistrurus catenatus* | (Britton et al., 2019) | 2012 | 26 (21 born in captivity) | NR | NR | NR | 13 | 5 | 0.19 | 5 | 0 | 5 | 0 | 13 | 0 | 0 | 5 | 5 (22 snakes died in total) |
| *Sistrurus catenatus* (3 snakes were brought into captivity) | (Tetzlaff et al., 2017) | 2013–2017 | 25 | 17 | 8 | 0.32 | 18 | 7 | 0.28 | NA | NA | NA | 0 | 18 | 0 | 7 | 0 | 6 (One snake was euthanized and 2 others died in captivity) |
| *Sistrurus catenatus ***  ***Not clear how long snakes were in captivity, if at all*** | (Allender et al., 2016b) | 2013–2014 | 184 | 162 | 22 | 0.12 | 173 | 11 | 0.06 | NA | NA | NA | 0 | 173 | 0 | 11 | 0 | 0 |
| *Sistrurus catenatus ****  ***Not clear how long snakes were in captivity, if at all*** | (Allender et al., 2016b) | 2013–2014 | 85 | NR | NR | NR | 67 | 18 | 0.21 | NA | NA | NA | 0 | 67 | 0 | 18 | 0 | 0 |
| *Sistrurus catenatus††*  ***Not clear how long snakes were in captivity, if at all*** | (Allender et al., 2015d) | 2009–2014 | 80 | NR | NR | NA | 64 | 13 | 0.17 | NR | NR | NR | 0 | 0 | 13 | 0 | 0 | 0 |
| *Sistrurus catenatus†††*  ***Not clear how long snakes were in captivity, if at all*** | (Allender et al., 2015d) | 2009–2014 | 50 | 50 | 0 | NA | NA | NA | NA | NA | NA | NA | 0 | 0 | 0 | 0 | 0 | 0 |
| ***Sistrurus miliarius*** | (Snyder and Sutton, 2020) | 2017–2018 | 10 | 10 | 0 | 0 | 10 | 0 | 0 | NA | NA | NA | 0 | 10 | 0 | 0 | 0 | 0 |
| *Sistrurus miliarius* | (Agugliaro et al., 2020) | 2017–2018 | 23 | NR | NR | NR | 14 | 9 | 0.39 | NA | NA | NA | 0 | 14 | 9 | 0 | 0 | 0 |
| ***Sistrurus miliarius*** | (Lind et al., 2019b)******** | 2015, 2017 | 252 | 219 | 33 | 0.13 | 88 | 40 | 0.16 | NR | NR | NR | 212 | 0 | 0 | 40 | 0 | 0 |
| ***Sistrurus miliarius*** | (Haynes et al. 2020) | 2016–2018 | 16 | 14 | 2 | 0.13 | 15 | 1 | 0.06 | NA | NA | NA | 0 | 15 | 0 | 1 | 0 | 0 |
| ***Sistrurus miliarius barbouri*** | (Cheatwood et al., 2003b) | 1997–1998 | ~600 | ~514 | 86 | 0.14 | NA | NA | NA | 5 | 0 | 5 | 0 | 0 | 0 | 5 | 0 | 0 |
| ***Sistrurus miliarius barbouri*** | (Lorch et al., 2016) | 2012 | 3 | 0 | 3 | 1 | 0 | 3 | 1 | 3 | 0 | 3 | 0 | 0 | 0 | 0 | 3 | 3 |
| *Sistrusrus miliarius* | (Lind et al., 2019a) | 2017 | 27 (Pregnant) | 16 | 11 | 0.41 | NR | 8 | NA | 2 | 0 | 2 | 0 | 0 | 0 | 6 | 2 | 3 |
| ***Storeria dekayi*** | (Fuchs et al., 2020) | 2018 | 2 | 1 | 1 | 0.5 | 1 | 1 | 0.5 | NA | NA | NA | 0 | 1 | 0 | 1 | 0 | 0 |
| *Storeria dekayi dekayi* | (Licitra et al., 2019)**††††** | 2015, 2017 | 1 | 0 | 1 | 1 | NR | NR | NR | 1 | 0 | 1 | 0 | 0 | 0 | 0 | 1 | 1 |
| ***Storeria dekayi*** | (Snyder and Sutton, 2020) | 2017–2018 | 15 | 15 | 0 | 0 | 15 | 0 | 0 | NA | NA | NA | 0 | 15 | 0 | 0 | 0 | 0 |
| ***﻿Storeria occipitomaculata*** | (McKenzie et al., 2019) | 2015–2016 | 3 | 2 | 1 | 0.33 | 2 | 1 | 0.33 | NA | NA | NA | 0 | 2 | 0 | 1 | 0 | 0 |
| *Thamnophis radix* | (Dolinski et al., 2014) | 2012 | 1 | 0 | 1 | 1 | 0 | 1 | 1 | 1 | 0 | 1 | 0 | 0 | 0 | 0 | 1 | 1 (Euthanized) |
| ***Thamnophis saurita saurita*** | (Fuchs et al., 2020) | 2018 | 9 | 8 | 1 | 0.11 | 8 | 1 | 0.11 | NA | NA | NA | 0 | 8 | 0 | 1 | 0 | 0 |
| ***Thamnophis saurita saurita*** | (Guthrie et al., 2016) | 2014 | 1 | 0 | 1 | 1 | NA | NA | NA | NA | NA | NA | 0 | 0 | 0 | 0 | 0 | 0 |
| ***Thamnophis saurita*** | (Haynes et al. 2020) | 2016–2018 | 19 | 14 | 5 | 0.26 | 18 | 1 | 0.05 | NA | NA | NA | 0 | 18 | 0 | 1 | 0 | 0 |
| ***Thamnophis sirtalis*** | (Fuchs et al., 2020) | 2018 | 2 | 2 | 0 | 0 | 2 | 0 | 0 | NA | NA | NA | 0 | 2 | 0 | 0 | 0 | 0 |
| ***﻿Thamnophis sirtalis*** | (McKenzie et al., 2019) | 2015–2016 | 8 | 3 | 5 | 0.63 | 4 | 4 | 0.5 | NA | NA | NA | 0 | 3 | 1 | 4 | 0 | 0 |
| ***Thamnophis sirtalis*** | (Snyder and Sutton, 2020) | 2017–2018 | 13 | 13 | 0 | 0 | 13 | 0 | 0 | NA | NA | NA | 0 | 13 | 0 | 0 | 0 | 0 |
| ***Thamnophis sirtalis*** | (Haynes et al. 2020) | 2016–2018 | 36 | 29 | 7 | 0.19 | 33 | 3 | 0.08 | NA | NA | NA | 0 | 33 | 0 | 6 | 0 | 0 |
| ***Thamnophis sirtalis sirtalis*** | (Guthrie et al., 2016) | 2014 | 4 | 4 | 0 | 0 | NA | NA | NA | NA | NA | NA | 0 | 0 | 0 | 0 | 0 | 0 |
| ***Thamnophis sirtalis sirtalis*** | (Licitra et al., 2019)**††††** | 2015, 2017 | 3 | 2 | 1 | 0.33 | NR | NR | NR | NR | NR | NR | 0 | 0 | 0 | 1 | 0 | 0 |
| ***Virginia valeriae*** | (McKenzie et al., 2019) | 2015–2016 | 3 | 2 | 1 | 0.33 | 2 | 1 | 0.33 | NA | NA | NA | 0 | 1 | 3 | 0 | 0 | 0 |
| ***Virginia valeriae*** | (Snyder and Sutton, 2020) | 2017–2018 | 7 | 7 | 0 | 0 | 6 | 1 | 0.14 | NA | NA | NA | 0 | 6 | 1 | 0 | 0 | 0 |

NA = Not Applicable (qPCR/PCR/Culture or histology not performed)

NR = Not Reported

† = This study is frequently cited to illustrate the potential impacts of ophidiomycosis. The authors report that this population exhibited a high frequency of substantial morphological deformities that were attributed to inbreeding. Of the 20 inferred or observed mortalities, fungal stomatitis was observed in one snake and another had a swollen eye. No other gross lesions were described. A fungus with similar morphology was cultured from oral or scale samples collected from three snakes and seen on histopathology of the lesions, but was not identified. To avoid discounting potential mortality from ophidiomycosis we have assumed that 20 mortalities in this study were caused by ophidiomycosis.

†† Samples collected in Illinois

††† Samples collected in Michigan

†††† Paper classified snakes only as “SFD Positive” and stated that criteria for specifying as such were “the presence of fungal dermatitis under histologic tissue analysis” OR “by positive PCR result”. The paper does not distinguish which sample used which criteria, therefore it was impossible to assign values for snakes under columns “qPCR/PCR/culture detection of Ophidiomyces ophiodiicola?” and “Presence of fungal hyphae in lesions?”. As either PCR or histology was performed, snakes that were classified as “SFD positive” in Licitra et al. 2019 were diagnosed as “Suspect Ophidiomycosis” in this table.

* all biopsy samples were presumed to have been taken from gross lesions

** museum specimens collected from the wild between 1999 and 2013

*** free-ranging snakes sampled in 2013 and 2014

**** 4 papers (McCoy et al 2017, Lind et al. 2018a, Lind et al, 2018b, and Lind et al 2019b, all appear to draw from a single dataset. We include the data summary presented in the most recent of these publications, to represent the most complete version of this dataset and to avoid recounting the same snakes.

**References** (additional to those already listed in the main text)

Agugliaro, J., Lind, C. M., Lorch, J. M., and Farrell, T. M. (2020). An emerging fungal pathogen is associated with increased resting metabolic rate and total evaporative water loss rate in a winter‐active snake. *Funct. Ecol.* 34, 486–496. doi:10.1111/1365-2435.13487.

Allender, M. C., Dreslik, M. J., Wylie, D. B., Wylie, S. J., Scott, J. W., and Phillips, C. A. (2013). Ongoing health assessment and prevalence of *Chrysosporium* in the Eastern Massasauga ( *Sistrurus catenatus catenatus*). *Copeia* 2013, 97–102. doi:10.1643/OT-12-004.

Allender, M. C., Junge, R. E., Baker-Wylie, S., Hileman, E., Faust, L., and Cray, C. (2015d). Plasma electrophoretic profiles in the Eastern Massasauga (*Sistrurus catenatus*) and influences of age, sex, year, location, and snake fungal disease. *J. zoo Wildl. Med.* 46, 767–773. doi:10.1638/2015-0034.1.

Allender, M. C., Hileman, E. T., Moore, J., and Tetzlaff, S. (2016a). Detection of *Ophidiomyces*, the causative agent of snake fungal disease, in the Eastern Massasauga (*Sistrurus catenatus*) in Michigan, 2014. *J. Wildl. Dis.* 52, 2015-12–333. doi:10.7589/2015-12-333.

Allender, M. C., Baker, S., Britton, M., and Kent, A. D. (2018). Snake fungal disease alters skin bacterial and fungal diversity in an endangered rattlesnake. *Sci. Rep.* 8, 1–9. doi:10.1038/s41598-018-30709-x.

Barber, D. M., Poole, V. A., Sanchez, C. R., Roady, P., and Allender, M. C. (2016). Snake fungal infection associated with *Fusarium* found in *Nerodia erythrogaster transversa* (Blotched Water Snake) in Texas , USA. 47, 39–42.

Britton, M., Allender, M. C., Hsiao, S.-H., and Baker, S. J. (2019). Postnatal mortality in neonate rattlesnakes associated with *Ophidiomyces ophidiicola*. *J. Zoo Wildl. Med.* 50, 672-677. doi: 10.1638/2018-0198. Burbrink, F. T., Lorch, J. M., and Lips, K. R. (2017). Host susceptibility to snake fungal disease is highly dispersed across phylogenetic and functional trait space. *Sci Adv* 3, e1701387. doi:: 10.1126/sciadv.1701387.

Dolinski, A. C., Allender, M. C., Hsiao, V., and Maddox, C. W. (2014). Systemic *Ophidiomyces ophiodiicola* Infection in a free-ranging Plains Garter Snake (*Thamnophis radix*). *J. Herpetol. Med. Surg.*, 7–10. doi:10.5818/1529-9651-24.1.7.

Fuchs, L. D., Tupper, T. A., Aguilar, R., Lorentz, E. J., Bozarth, C. A., Fernandez, D. J., et al. (2020). Detection of *Ophidiomyces ophiodiicola* at two mid-Atlantic natural areas in Anne Arundel County, Maryland and Fairfax County, Virginia, USA.

Glorioso, B. M., Waddle, J. H., Green, D. E., and Lorch, J. M. (2016). First documented case of snake fungal disease in a free-ranging wild snake in Louisiana. *Southeast. Nat.*, 15: 4–6.

Guthrie, A. L., Knowles, S., Ballmann, A. E., and Lorch, J. M. (2016). Detection of snake fungal disease due to *Ophidiomyces ophiodiicola* in Virginia, USA. *J. Wildl. Dis.* 52, 143–149. doi:10.7589/2015-01-007.

Haynes, E., Chandler, H. C., Stegenga, B. S., Adamovicz, L., Ospina, E., Zerpa-Catanho, D., Stevenson, D. J. and Allender, M. C., 2020. Ophidiomycosis surveillance of snakes in Georgia, USA reveals new host species and taxonomic associations with disease. *Sci. Rep.* 10: 15362. doi:10.1038/s41598-020-67800-1.

Hill, A. J., Leys, J. E., Bryan, D., Erdman, F. M., Malone, K. S., Russell, G. N., et al. (2018). Common cutaneous bacteria isolated from snakes inhibit growth of *Ophidiomyces ophiodiicola*. *Ecohealth* 15, 109–120. doi:10.1007/s10393-017-1289-y.

Last, L. A., Fenton, H., Gonyor-mcguire, J., Moore, M., and Yabsley, M. J. (2016). Snake fungal disease caused by *Ophidiomyces ophiodiicola* in a free-ranging mud snake (*Farancia abacura*). *J Vet Diagn Invest* 28, 709-713. doi: 10.1177/1040638716663250.

Licitra, D., Quinn, D. P., Reeder, J. E., Gavitt, T., Dickson, J., Hess, B., et al. (2019). Snake fungal disease in Colubridae snakes in Connecticut, USA in 2015 and 2017. *J. Wildl. Dis.* 55, 658. doi:10.7589/2018-04-100.

Lind, C. M., Clark, A., Smiley-Walters, S. A., Taylor, D. R., Isidoro-Ayza, M., Lorch, J. M., et al. (2019a). Interactive effects of food supplementation and snake fungal disease on pregnant Pygmy Rattlesnakes and their offspring. *J. Herpetol.* 53, 282. doi:10.1670/18-147.

Lind, C. M., Lorch, J. M., Moore, I. T., Vernasco, B. J., and Farrell, T. M. (2019b). Seasonal sex steroids indicate reproductive costs associated with snake fungal disease. *J. Zool.* 307, 104–110. doi:10.1111/jzo.12628.

McKenzie, J. M., Price, S. J., Fleckenstein, J. L., Drayer, A. N., Connette, G. M., Bohuski, E., et al. (2019). Field Diagnostics and seasonality of *Ophidiomyces ophiodiicola* in wild snake populations. *Ecohealth* 16, 141–150. doi:10.1007/s10393-018-1384-8.

Price, S. J., Oldham, C. R., Boys, W. M., and Fleckenstein, L. J. (2016). First record of snake fungal disease in Kentucky. *J. Ky. Acad. Sci.* 76, 47–49.

Ravesi, M. J., Tetzlaff, S. J., Allender, M. C., and Kingsbury, B. A. (2016). Notes of the northeastern detection of snake fungal disease from a *Lampropeltis triangulum* (Eastern Milksnake) in northern Michigan. 2001–2004. *Northeastern Nat* 23, doi:10.1656/045.023.0310

Snyder, S. D., and Sutton, W. B. (2020). Prevalence of *Ophidiomyces ophiodiicola*, the causative agent of snake fungal disease, in the Interior Plateau Ecoregion of Tennessee, USA. *J. Wildl. Dis.,* doi: 10.7589/2019-04-109
